# Endotoxin removal by the OXIRIS filter for cardiogenic shock requiring veno-arterial extra-corporeal life support: the ECMORIX randomized controlled trial

**DOI:** 10.1186/s13613-025-01499-z

**Published:** 2025-07-08

**Authors:** Maxime Nguyen, Marvin Alvarez, Corentin Evezard, Vivien Berthoud, Damien Leleu, Jean-Paul Pais-De-Barros, Olivier Bouchot, Osama Abou-Arab, Belaid Bouhemad, David Masson, Thomas Gautier, Pierre-Grégoire Guinot

**Affiliations:** 1 Department of Anesthesiology and Intensive Care, Dijon University Hospital, Dijon, France; 2 University of Burgundy, Dijon, France; 3 Center for Translational and Molecular Medicine (CTM), Lipness Team, INSERM UMR1231, Dijon, France; 4 Laboratory of Clinical Chemistry, Dijon University Hospital, Dijon, France; 5 Lipidomic Facility, Université de Bourgogne, Dijon, France; 6 Department of Cardiac Surgery, Dijon University Hospital, Dijon, France; 7 Department of Anesthesiology and Critical Care Medicine, Amiens University Hospital, Amiens, France

**Keywords:** Endotoxemia, Cardiogenic-shock, ECMO, Translocation, Inflammation, Critical care

## Abstract

**Background:**

Most severe cardiogenic shock requires veno-arterial extracorporeal membrane oxygenation (VA-ECMO). The OXIRIS filter, has shown potential in reducing lipopolysaccharide (LPS) levels. Our objective was to compare the efficacy of the OXIRIS filter versus the ST-150 filter in reducing LPS plasma concentration. We hypothesized that the OXIRIS filter would reduce the endotoxin burden.

**Methods:**

We conducted an open-label randomized prospective study in the cardiac intensive care unit of Dijon University Hospital. Forty patients with refractory cardiogenic shock requiring VA-ECMO and renal replacement therapy (RRT) were randomized to receive either OXIRIS filter or ST-150 filter. Blood samples were collected at multiple time points. The primary outcome was LPS mass (measured 24 h after the initiation of treatment). Secondary outcomes included LPS activity, cytokine levels, and clinical outcomes.

**Results:**

20 patients were allocated to each group and analyzed. LPS plasma concentrations were not different between the OXIRIS filter and ST-150 filter groups at H24 (599 pmol/ml of esterified 3-OH fatty acids [450;734] vs 520 [456;835], p = 0.983) or when analyzing all time-points by linear mixed modelling (538 [469;723] vs 507 [434;671] at H26, 576 [513;614] vs 624 [503;724] at H48 and 632 [513;660] vs 586 [538;776] at H72, p = 0.882). No significant between groups differences were found in LPS activity, inflammation markers (IL-6, TNF-α, IL-10, MCP-1), SOFA scores, VIS scores, or 28-day mortality (13 (65%) vs 9 (45%) p = 0.21). There was high variability in LPS concentrations, suggesting heterogeneity in endotoxemia.

**Conclusions:**

In patients with cardiogenic shock supported by VA-ECMO and requiring renal replacement therapy, we could not evidence any reduction in LPS blood concentration in patients treated with treatment with OXIRIS filter in comparison to ST-150 filter. Further research is required to confirm these findings and optimize endotoxin removal in this population.

*Trials Registration:* NCT04886180. Registered 10 May 2021.

**Graphical Abstract:**

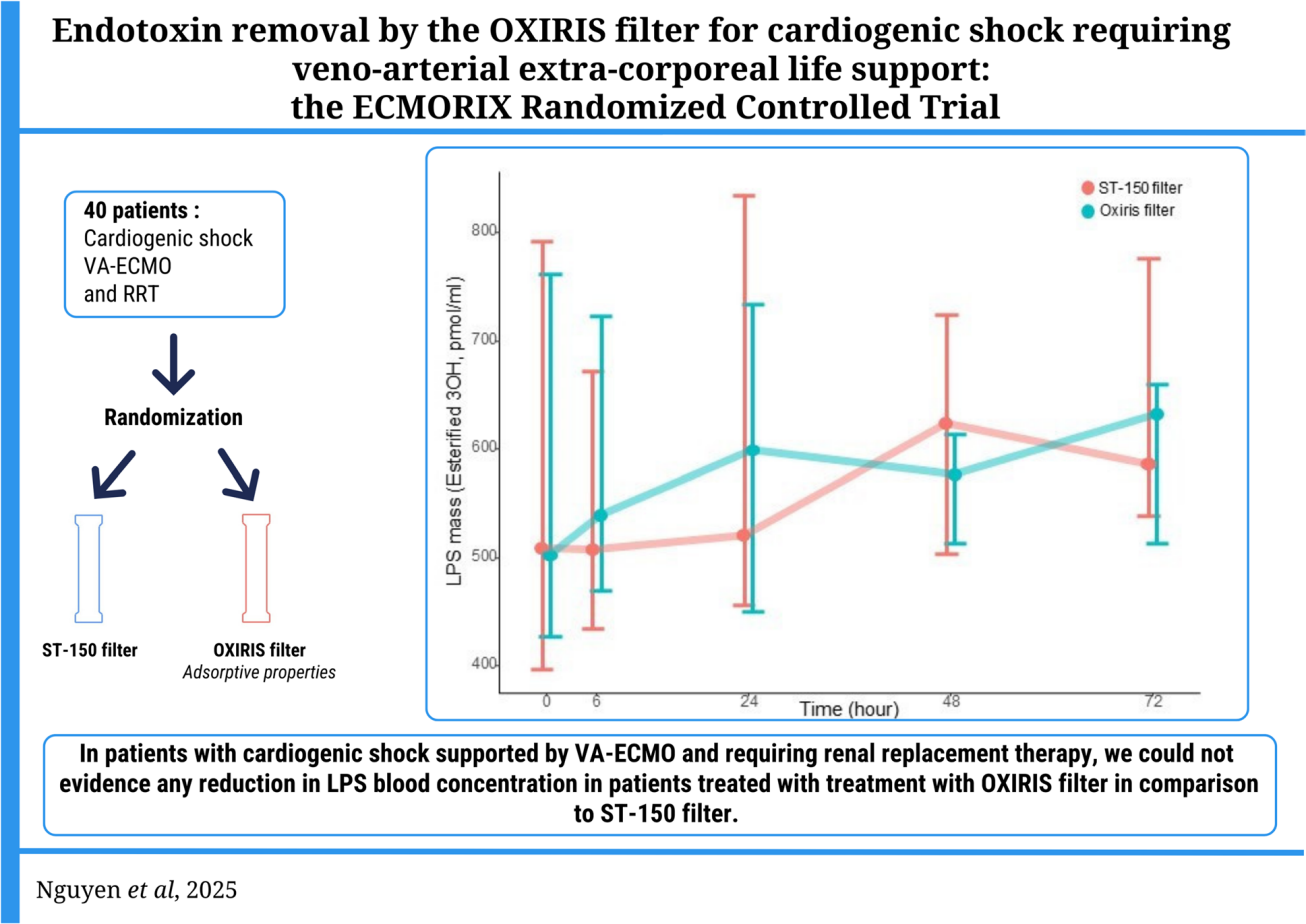

**Supplementary Information:**

The online version contains supplementary material available at 10.1186/s13613-025-01499-z.

## Background

Cardiogenic shock refers to the inability of the heart to ensure adequate tissue perfusion [1]. When cardiac function remains insufficient to maintain adequate tissue perfusion despite adequate treatment with vasopressors and inotropes, circulatory function might be mechanically supported by veno-arterial extracorporeal membrane oxygenation (VA-ECMO) [2]. VA-ECMO support is required in up to 20% of cardiogenic shock patients, with this proportion varying significantly depending on the underlying etiology [3, 4]. Patients with cardiogenic shock might develop ischemic organ injury due to insufficient oxygen delivery. This phenomenon is thought to increase with shock severity (ie. to occur especially in patients with stage D or E cardiogenic shock based on the society for cardiovascular angiography & interventions (SCAI) classification) [5]. Additionally, acute kidney injury represents a major concern in this population, affecting up to 80% of patients, with approximately 20% requiring renal replacement therapy [6, 7]. VA-ECMO, by restoring systemic blood flow, enables organ reperfusion. However, reperfusion of ischaemic tissues induces an exacerbation of cellular dysfunction and death, resulting in ischaemia–reperfusion injury [8]. Injury of the digestive tract promotes disruption of the gut barrier, allowing endotoxin translocation from the gut lumen to the blood [9]. Intestinal fatty acid binding protein (I-FABP) is a marker of enterocyte damage, citrulline is a marker of enterocyte mass [10] and glucagon-like peptide 1 has been described as a marker of mucosal integrity [11, 12]. As endotoxin is a strong inducer of inflammation through TLR-4 activation [13], endotoxaemia might increase circulatory failure by enhancing vasoplegia and myocardial dysfunction, thus contributing to the evolution of multi-organ failure and death [14]. Altogether, risk stratification based on cardiogenic shock severity should enable to select patients with endotoxemia. The OXIRIS renal replacement therapy (RRT) filter (BAXTER, Deerfield, USA) is coated with polyethyleneimine and has been demonstrated to adsorb endotoxin ex vivo [15].

Our objective was to compare the efficacy of the OXIRIS filter versus the ST-150 filter in reducing LPS levels in patients with refractory cardiogenic shock, requiring VA-ECMO and renal replacement therapy. LPS mass and activity provide different and complementary information. Human plasma has the ability to inactivate LPS. Thus, a decay in LPS activity might be due either to LPS elimination or to LPS inactivation. LPS mass is not modified par inactivation phenomenon [16]. Thus, in the context of evaluating extra-corporeal LPS removal, LPS mass appear to be more relevant (as elimination refers to a decay in mass). We hypothesized that the OXIRIS filter would reduce the endotoxin burden in this high-risk population, thus helping in the resolution of inflammation, and improving vasoplegia, organ failure, and survival.

## Material and methods

### Ethics

This was a randomized prospective study conducted in the Dijon Cardiovascular Intensive Care Unit (CICU). This research was approved by the institutional review board (CPP Sud-ouest et Outre-mer II, France; ref. 2021-A00097-34). Informed consent was obtained from all patients or their next of kin prior to inclusion. Patients were included between May 2021 and March 2023. Inclusion criteria were: age ≥ 18 years, with refractory cardiogenic shock treated by VA-ECMO for less than 12 h, and requiring renal replacement therapy (RRT). Indications for RRT were left to attending physician and follow our standard protocol (metabolic acidosis, fluid removal, azotemia and hyperkaliemia). Exclusion criteria were: patients with severe hemorrhage, patients not affiliated with health insurance, under legal guardianship, pregnant or breastfeeding. The present report was drafted in accordance with the CONSORT statement [17].

### Protocol

Before RRT initiation, patients were randomized by the investigator on a dedicated platform (Cleanweb^™^) to be treated either with an OXIRIS filter (BAXTER, Deerfield, USA) or a ST-150 filter. RRT was initiated on PRISMAFLEX systems or PRISMAX systems (BAXTER, Deerfield, USA) devices. RRT was incorporated into the VA-ECMO system and all patients received CVVH. There is no consensus on the timing and RRT modalities in this population. The management of RRT (indication, duration, and parameters) as well as cardiogenic shock was handled by the attending physician, following standard care protocols, as previously described [18]. Only RRT filters used during the 72 h after inclusion were impacted by the protocol. RRT set-up was performed by the patient's assigned nurse. Although the caregiver was not blinded to the treatment group, data collection, outcomes, and measurements were recorded and performed by research staff blinded to the allocation group. Plasma samples were taken from the RRT circuit before the filter at RRT initiation (H0), and at 6 h (H6), 24 h (H24), 48 h (H48), and 72 h (H72) after RRT initiation. Demographic and clinical data were collected during the ICU stay [18]. All biological assay were conducted on pre-filter sample. LPS mass, activity and cytokines were measured on both pre- and post-filter sample. Society for Cardiovascular Angiography and Interventions (SCAi) classification was calculated a posteriori.

### Biological measurement

Blood was collected in EDTA tubes and centrifuged (2000 g, 4 min, 4 °C). Plasma was then stored at −80 °C before analysis. At the end of the study, these samples were used for biological assays. Assays were performed blinded to the treatment group. LPS mass concentration was determined using liquid chromatography coupled with mass spectrometry. LPS mass is given as pmol/ml of esterified 3-OH fatty acids (esterified 3-hydroxy-fatty acids with chain lengths ranging from 10 to 18 carbons obtained by subtracting total 3-hydroxy fatty acids and non-esterified 3-hydroxy fatty acids). LPS activity was measured by the LAL (Limulus amebocyte lysate) assay (HycultBiotech Inc., Wayne, USA). Citrulline (Abbexa, Cambridge, UK), Intestinal fatty acid binding protein (I-FABP) (HycultBiotech Inc., Wayne, USA) and glucagon like peptide 1(GLP-1) (Mercodia, Uppsala, Sweden) were measured by ELISA. For all these kits, the manufacturer's instructions were followed. High-density lipoprotein cholesterol (HDLc) and low-density lipoprotein cholesterol (LDLc) concentrations were measured on the Atellica system using dedicated kits (Siemens-France). Cytokines were measured using a Luminex^®^ Human Magnetic assay (Merck, Darmstadt, Germany), following the manufacturer's instructions. According to the manufacturer’s instructions, plasma with hemolysis were excluded for LAL, citrulline and I-FABP measurements.

### Outcomes

The primary outcome was LPS mass measured 24 h after RRT initiation. The secondary outcomes were as follows: LPS activity measured by LAL, inflammation assessed by cytokine concentrations, vasoplegia assessed by the vasoactive-inotropic score (VIS) [19], organ failure assessed by SOFA, digestive distress assessed by I-FABP, citrulline, and GLP-1, and 28-day mortality [18].

### Calculation

Because the pre-filter sample was drawn after the pre-blood pump (ie. after pre-dilution), concentrations were corrected as follow [X]_preC_ = [X]_pre_*[(Q_s_ + Q_pre_)/Q_s_].

Parameters with value out of the limit of detection were not corrected for haemodilution (value left at the limit of detection) to avoid a bias related to dilution factor (All patients at the limit of detection for a given parameter would be ranked by predilution for this parameter). There was no value out of the limit of detection for LPS mass (primary outcome). The limit of detection values for LPS activity were [0.04;10] EU/ml.

### Statistical analysis

We hypothesized that patients treated with the OXIRIS filter would have a decrease of LPS mass greater than 35% at H24 compared to patient treated with the ST-150 filter (Hypotheses 1). Based on previous data regarding LPS measurement in patients undergoing cardiac surgery, we calculated a sample size of 34 patients (17 per group) with an alpha risk of 5% and a power of 80%, using a two-tailed test [20]. Due to the risk of incomplete data and early mortality (high risk population), the sample size was increased to 40 patients (20 patients per group). Quantitative data are presented as mean and standard deviation or median and interquartile range, depending on their distribution. Qualitative data are presented as frequency and percentage. We performed an intention-to-treat analysis. Patients were separated in two groups based on randomisation. These groups were compared. Primary outcome was tested based on the pre-specified hypothesis (H1). Quantitative data were compared using the Kruskal–Wallis non parametric test, and qualitative data were compared using the Chi-square test or Fisher's exact test, depending on whether the validity conditions were met. Survival data were represented on a Kaplan Meier plot and were compared with a log-rank test. Longitudinal data were processed using linear mixed modelling (LMM), variables of interest were modeled depending of group allocation and time, patient identifiers were used as random intercept. Kenward-Roger's approximation of the degrees of freedom was used. Normality of random effects and model residuals distributions were graphically checked. Some variables (LPS activity, GLP-1, citrulline, IL-6, IL-10, TNF-α, MCP-1, VIS score) were log-transformed to improve model validity. Because some slight deviations from normality were tolerated, concordance of LMM with repeated non-parametric comparison at each time point was checked. Statistical significance was defined as a *p*-value of less than 0.05. Missing data were omitted from the analysis. Statistical analysis was performed using R (version 4.3.0).

## Results

### Population and baseline characteristics

**Fig. 1:**
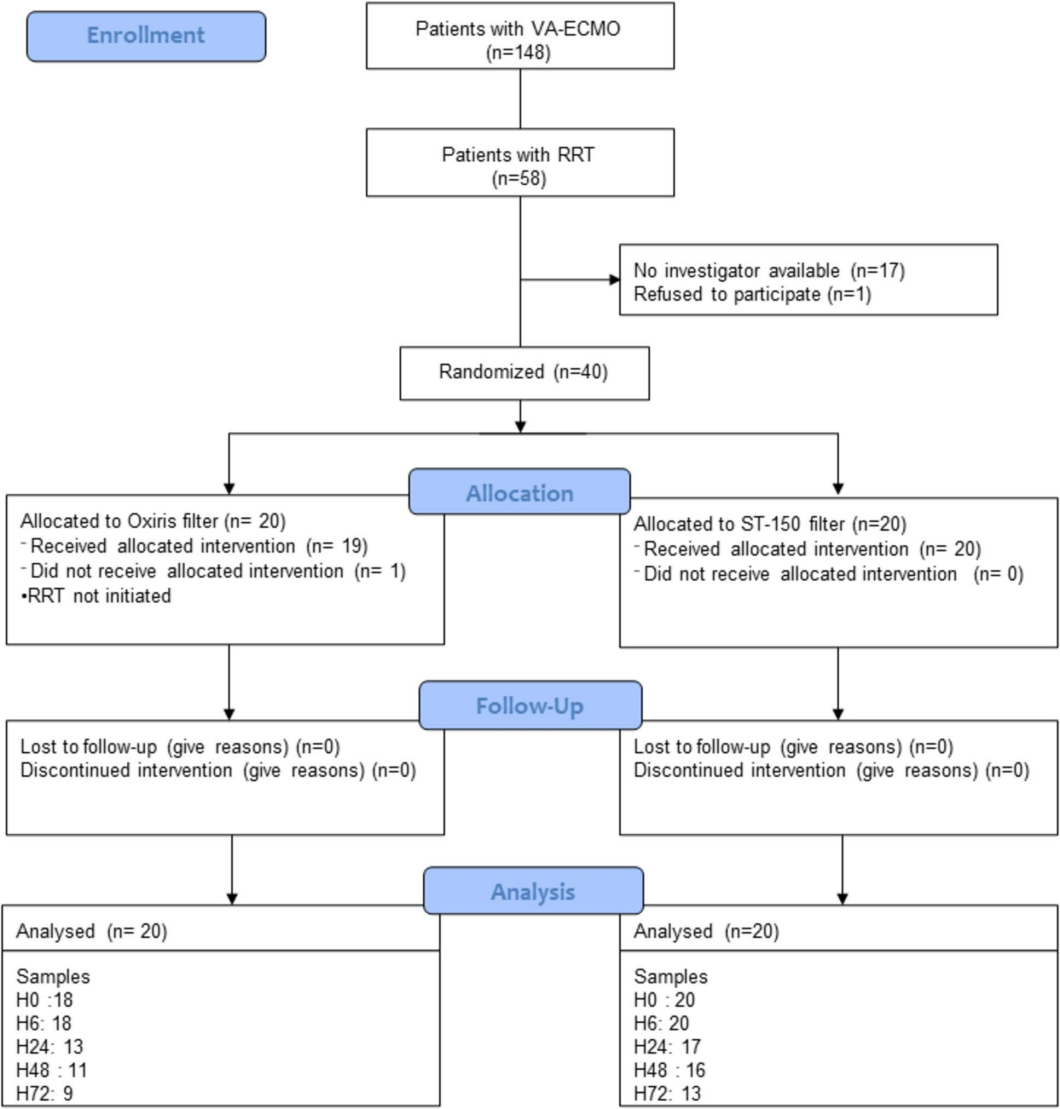
Flow chart of the study. *VA-ECMO* Veno-arterial extracorporeal membrane oxygenation, *RRT* renal replacement therapy

Forty patients were randomized and analyzed; one patient allocated to the OXIRIS filter group did not receive the intervention (Fig. 1). The number of sample available at each time point is given in Fig. 1. Some measurements were missing due to early death (6 patients died between H6 and H24, 3 between H24 and H48 and 2 between H48 and H72) and omission to sample the patient at the given time-point. Baseline characteristics are provided in Table 1. The mean SAPS II was 71 (± 21). RRT and VA-ECMO parameters are given in supplementary Table 1. The number of filters per patient used within the first 72 h was similar between groups (1 [1;2] vs 1 [1;2], p = 0.396) Patients were similar in terms of age, sex, medical history, and severity.

**Table 1:** Baseline characteristics according to group allocation

	ST-150 filter N = 20	OXIRIS filter N = 20
Age (years)	64.0 [56.8;64.8]	65.0 [59.5;67.8]
Sex (W)	6 (30%)	12 (60%)
BMI (Kg/m^2^) (n = 38)	25.2 [21.6;28.6]	27.7 [24.3;33.0]
Medical history		
Myocardial infarction	7 (35%)	7 (35%)
Peripheral atherosclerosis	3 (15%)	4 (20%)
Stroke	0 (0%)	1 (5%)
Hypertension	8 (40%)	12 (60%)
Dyslipidemia	10 (50%)	9 (45%)
Diabetes	5 (25%)	9 (45%)
Cancer	1 (5%)	1 (5%)
Malignant hemopathy	3 (15%)	0 (0%)
Chronic respiratory failure	2 (10%)	3 (15%)
Chronic renal failure	6 (30%)	2 (10%)
Chronic cardiac failure (n = 39)	8 (40%)	6 (31.6%)
Etiology		
Myocardial infarction	5 (25%)	9 (45%)
Post cardiac arrest	3 (15%)	4 (20%)
Post cardiotomy	7 (35%)	1 (5%)
Septic	2 (10%)	2 (10%)
Toxic	0 (0%)	1 (5%)
Obstructive	1 (5%)	1 (5%)
Other	2 (10%)	2 (10%)
SCAi classification		
C	2 (10%)	2 (10%)
D	11 (55%)	9 (45%)
E	7 (35%)	9 (45%)
Severity		
SAPS II score	69.0 [62.8;81.5]	58.5 [51.0;84.0]
VIS score	64.1 [36.9;119]	44.0 [11.7;108]
Lactatemia (mmol/L)	5.25 [3.95;10.2]	6.60 [4.15;9.00]
Creatininemia (µmol/L)	138 [111;211]	130 [104;205]
AST (UI/L)	468 [135;1200]	1259 [234;2248]
ALT (UI/L)	164 [47.8;323]	328 [56.2;1598]
Bilirubin (µmol/L)	19.5 [10.8;26.5]	13.5 [9.00;17.2]
Troponin (ng/L) (n = 39)	9633 [4573;35863]	13343 [3826;189095]

*BMI* Body mass index, *SAPS* Simplified acute physiology score, *VIS* Vasoactive-inotropic score, *ALT* alanine amino transferase, *AST* Aspartate amino transferase, *SCAi* Scociety for Cardiovascular Angiography and Interventions

n = 40 for all data unless specified otherwise

### Treatment with OXIRIS filter did not lower LPS concentrations

**Fig. 2:**
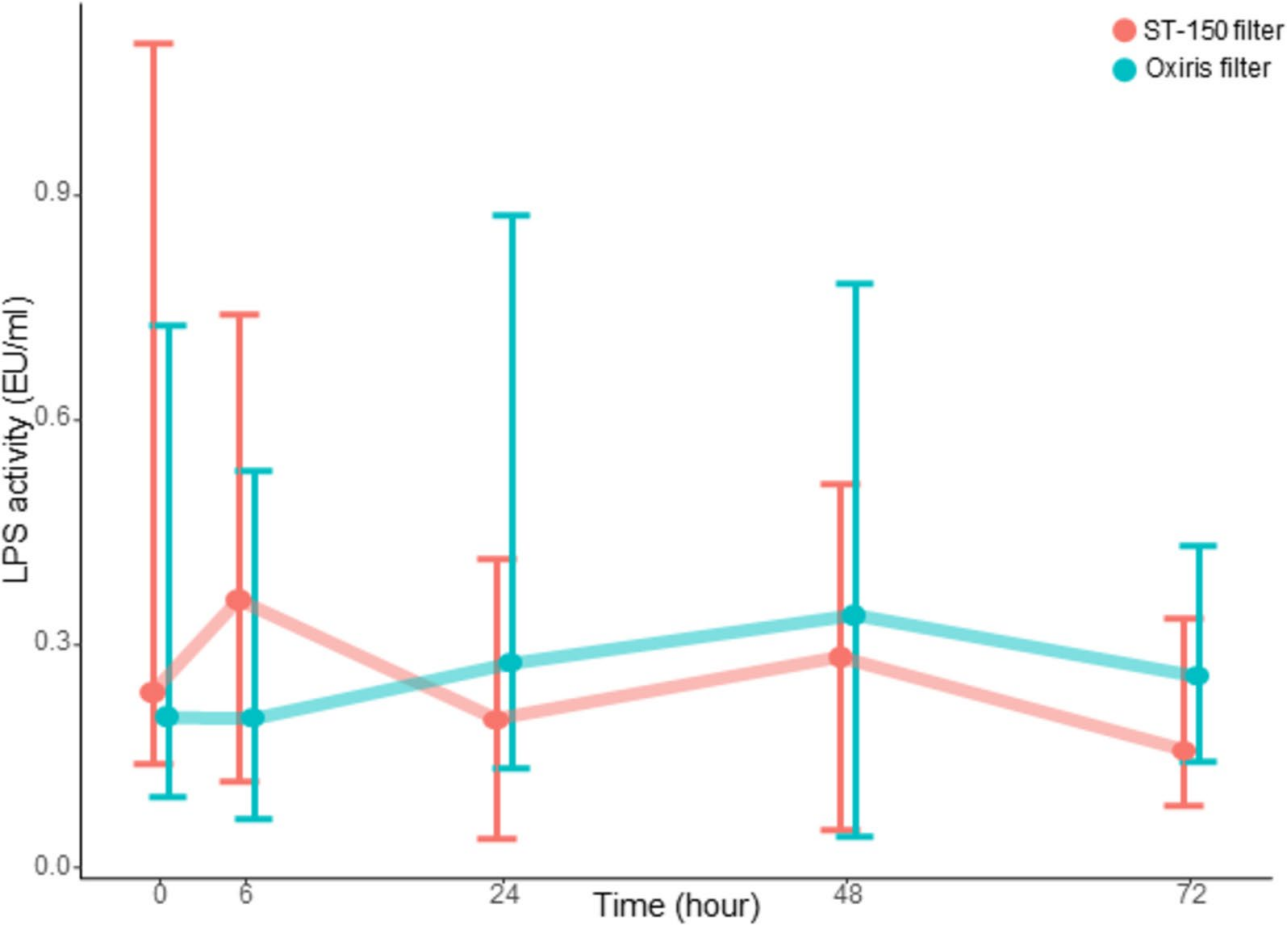
Lipopolysaccharides activity depending on group allocation. Results are represented as median an [Q1;Q3]; there were no significant between group differences by linear mixed modelling. *LPS* Lipopolysaccharides

LPS plasma concentrations were not different between the OXIRIS filter and ST-150 filter groups at H24 (599 [450;734] vs 520 [456;835], p = 0.983) (Sup Fig. 1 and 2). There was no difference in LPS activity between the groups (Fig. 2, Sup Fig. 2). A post-hoc analysis conducted in patients with high LPS mass (8 with OXIRIS filter vs 11 with ST-150 filters) did not evidence any overall difference in LPS mass or activity between groups (by mixed linear modelling) (Sup Fig. 3).

### Treatment with OXIRIS filter did not reduce inflammation or improve clinical outcomes

**Fig. 3:**
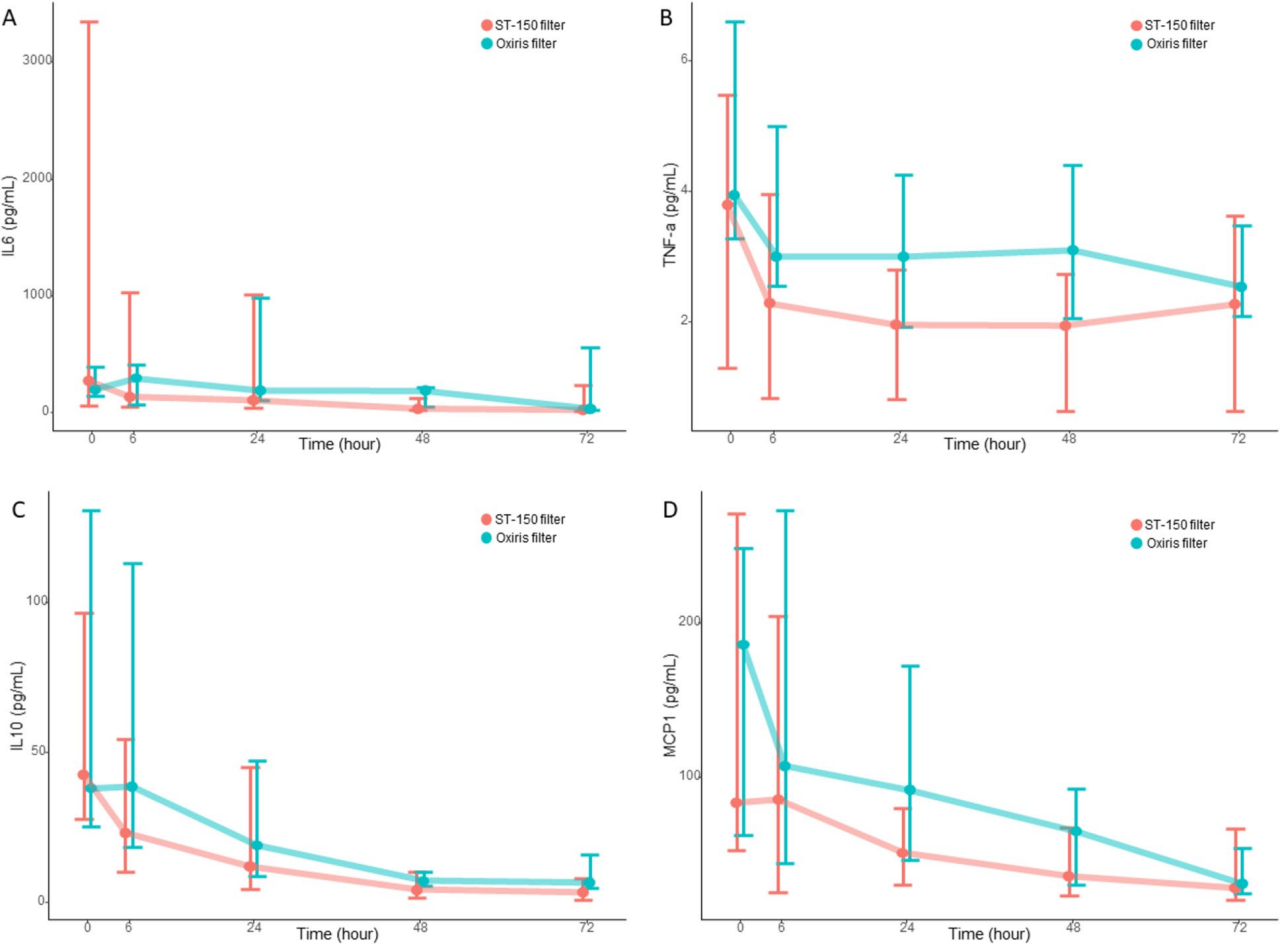
Cytokines plasma concentration depending on group allocation. Results are represented as median an [Q1;Q3]; there were no significant between group differences or time effect by linear mixed modelling. *IL* Interleukin, *TNF* Tumor necrosis factor, *MCP* Monocyte chemoattractant protein

**Fig. 4:**
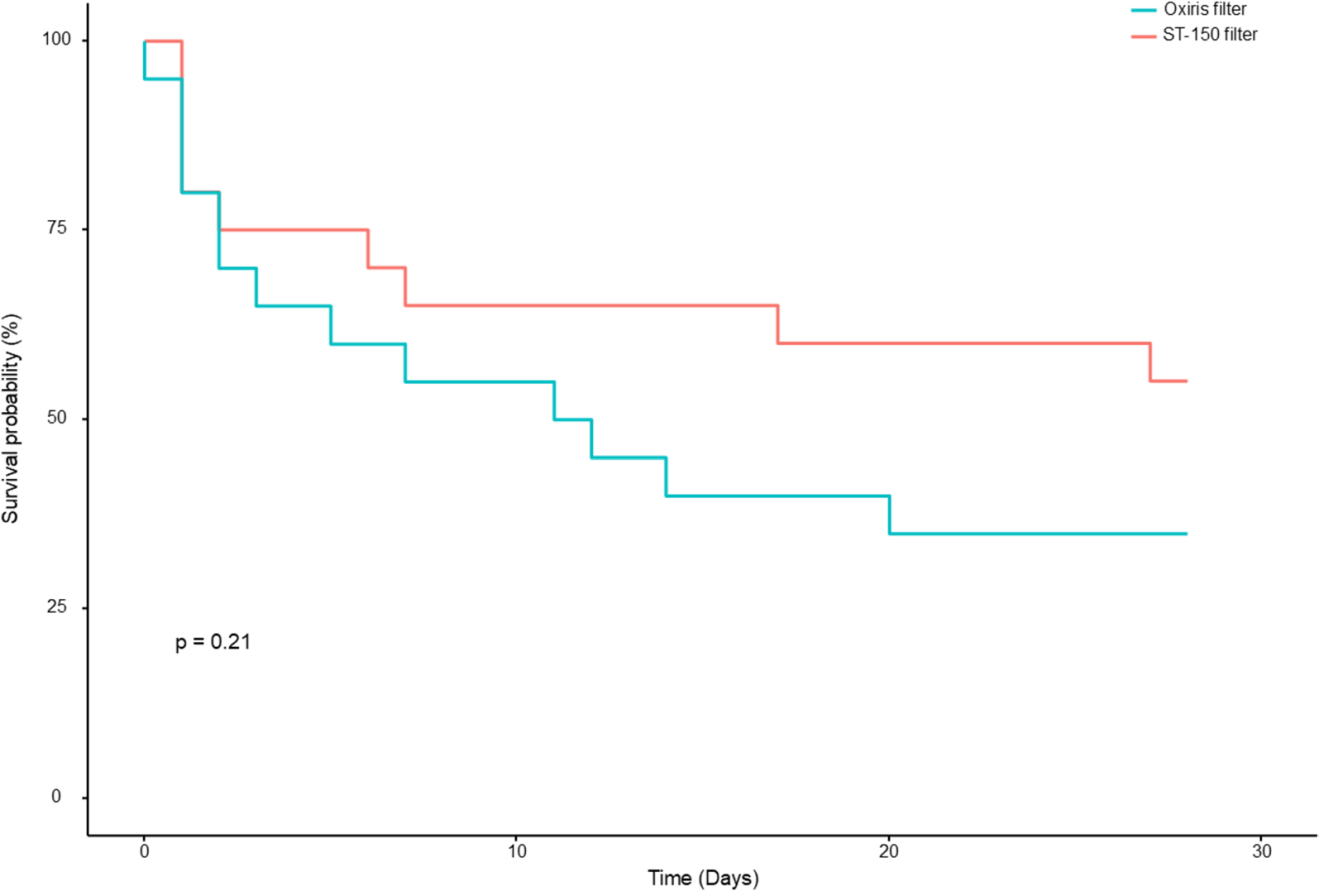
Survival probability according to group allocation. p refers to between groups comparison (Log-rank test)

Patients treated with the OXIRIS filter did not have lower levels of cytokines compared to patients treated with ST-150 (IL-6, TNF-α, IL-10, MCP-1) (Fig. 3). Additionally, these patients did not exhibit reduced biomarkers of digestive distress (I-FABP, Citrulline, GLP-1) at any time point (Table 2), lower organ failure measured by SOFA scores or vasoplegia assessed by VIS scores (Table 3). Lastly, there was no difference in 28-day mortality between the groups (13 (65%) vs 9 (45%) p = 0 0.21 in the OXIRIS filter and ST-150 filter group respectively) (Fig. 4).

**Table 2:** Biomarker of digestive suffering to group allocation

	ST-150 filter N = 20	OXIRIS filter N = 20	p-value
I-FABP (pg/ml)*			
H0 (= 33)	7500 [1853;7500]	4023 [1806;7500]	0.488
H6 (n = 35)	2554 [892;6791]	1916 [656;3630]	
H24 (n = 28)	335 [60.7;1426]	1109 [298;2193]	
H48 (n = 25)	45.8 [0.00;466]	0.00 [0.00;186]	
H72 (n = 22)	125 [0.00;328]	62.5 [0.00;428]	
Citrulline (ng/ml)			
H0 (n = 33)	42.6 [16.5;67.9]	28.4 [13.4;90.8]	0.906
H6 (n = 35)	23.0 [8.56;75.4]	33.4 [17.5;64.9]	
H24 (n = 28)	21.3 [10.1;52.3]	44.9 [0.00;91.2]	
H48 (n = 25)	20.3 [7.38;51.0]	9.94 [1.50;34.5]	
H72 (n = 22)	16.1 [0.00;59.5]	16.2 [0.00;41.7]	
GLP-1 (pg/ml)*			
H0 (n = 37)	45.0 [22.2;59.6]	60.3 [40.7;83.5]	0.448
H6 (n = 38)	33.8 [18.7;64.4]	46.2 [25.9;69.7]	
H24 (n = 30)	32.2 [25.4;58.0]	65.4 [39.1;72.5]	
H48 (n = 27)	45.4 [28.3;70.9]	63.5 [34.2;71.2]	
H72 (n = 22)	66.5 [29.1;85.1]	60.7 [42.2;77.0]	

*I-FABP* Intestinal fatty acid binding protein, *GLP-1* Glucagon like peptide 1

Linear mixed model was used for comparisons. p refers to between groups comparison. *Indicate a significant effect of time. n refers to the number of observations

**Table 3:** Vaso-inotropic score and sequential organ failure assessment score according to group allocation

	ST-150 filter N = 20	OXIRIS filter N = 20	p-value
VIS*			0.412
H0 (n = 40)	64.1 [36.9;119]	44.0 [11.7;108]	
H6 (n = 40)	78.0 [24.3;104]	31.0 [10.3;114]	
H24 (n = 34)	24.1 [8.78;55.4]	27.1 [5.00;72.8]	
H48 (n = 30)	7.50 [1.50;32.5]	5.00 [0.00;25.4]	
H72 (n = 28)	0.00 [0.00;5.75]	0.00 [0.00;3.00]	
SOFA score			0.240
H0 (n = 40)	11.2 (2.85)	10.2 (3.31)	
H24 (n = 34)	11.6 (3.10)	11.2 (3.66)	
H48 (n = 27)	11.2 (3.36)	8.85 (2.67)	
H72 (n = 27)	9.36 (2.87)	9.38 (2.96)	

*VIS* vasoactive-inotropic score, *SOFA* Sequential organ failure assessment

Linear mixed model was used for comparisons. p refers to between groups comparison. * refers to significant time variation. n refers to the number of observations

## Discussion

In patients with cardiogenic shock supported by VA-ECMO and requiring renal replacement therapy, within the sample size of this study, treatment with OXIRIS filter was not associated with lower LPS plasma concentrations compared to treatment with the ST-150 filter. We could not evidence a reduction in inflammatory biomarkers, an improvement in organ failure, vasoplegia, or survival.

Our findings contrast with previous studies that reported a reduction of LPS activity ex vivo using the OXIRIS filter [15, 21]. Several hypotheses might explain these differences. First, in ex vivo studies, a unique dose of LPS was used, whereas in patients with loss of gut barrier function, it is likely that continuous LPS translocation occurs due to digestive ischaemia–reperfusion and loss of barrier function. This continuous translocation may result in varying amounts of LPS and potential saturation of the membrane. Second, the ex vivo studies only measured LPS activity, whereas in plasma, LPS might be neutralised by binding to lipoproteins [22, 23]. A reduction in LPS activity does not necessarily imply LPS elimination but might be related to LPS inactivation [16]. Thus, measuring both LPS mass and activity is necessary to differentiate elimination from inactivation. Since we measured LPS mass and did not observe any difference between the two groups, our results challenge in-vitro studies evaluating only the LPS activity [15].

In patients with cardiogenic shock, LPS translocation is thought to be a driver of systemic inflammation, contributing to adverse outcomes [5]. However, data on LPS translocation in cardiogenic shock patients are scarce [24]. Data from septic patients suggest that LPS dose likely influences the response to blood purification [25]. By recruiting patients with the most severe cardiogenic shock, we aimed to select those with the highest risk of digestive ischaemia–reperfusion and LPS translocation [26]. Despite focusing on the most critically ill cardiogenic shock patients, we observed substantial variability in LPS plasma concentrations at study inclusion. The wide interquartile range of these concentrations suggests a high degree of heterogeneity within our patient cohort. This observation aligns with findings from Lee et al., who observed that global endotoxemia activity was low, with only 4% of patients with VA-ECMO expressing high endotoxin activity. However, they observed that the worst outcomes were in patients with higher endotoxemia levels [27]. Those observations, suggest that a more tailored treatment approach may be necessary to target those patients most likely to benefit from endotoxin removal [28].

In our cohort, we observed that LPS activity and cytokine concentrations were also highly variable. This finding suggests that the severity of cardiogenic shock alone may not be a sufficient criterion for initiating haemoadsorption therapy. Inflammation during cardiogenic shock is multifactorial, involving both danger and pathogen-associated molecular patterns. The host response to cardiogenic shock is poorly studied [29]. Our results indicate that the inflammatory profile in cardiogenic shock probably differs from that of septic shock, despite both conditions being associated with critical illness. This distinction highlights the need for a more nuanced approach to patient selection for haemoadsorption therapy in cardiogenic shock. Further research is required to identify clinical or biological markers to better guide the selection of these patients. As in sepsis, it is likely that patients with cardiogenic shock exhibit highly heterogeneous responses to the insult, with several endotypes potentially describable [30, 31]. Implementing therapies that aim to regulate the host response (e.g., LPS removal) will likely depend on these endotypes. Further investigation into the host response to cardiogenic shock may be a first step towards precision medicine in this population.

We can discuss several limitations. Although the sample size was powered, it remains relatively small, and the absence of between-group differences might relate to low power. The power calculation was based on LPS mass estimated by C14 3OH fatty acids measurement. The LPS mass analyzed here are C10 to C18 3OH esterified fatty acids. The number of expected deaths in cardiogenic shock has been underestimated, and as many patients died early after randomization, we chose not to exclude patients with limited exposure to the intervention (i.e., less than 24 h). This decision was made to avoid further reducing the statistical power of the analysis. The sample size in our study was similar to those used in previous studies evaluating cytokine and/or LPS removal with RRT, and it remained within the calculated patient requirements. Nevertheless, the study was probably underpowered to detect differences in both primary and secondary outcomes. The open-label nature of the study introduces a potential performance bias. This is a single-centered study which limits the generalizability of the findings for other cardiac ICUs. Despite randomization, post-cardiotomy and sex ratio seemed unbalanced between groups and may have acted as confounders. The use of local anticoagulation citrate may affect LPS measurements [32]. The inclusion of patients with a history of hemopathies may also have introduced a bias, as this is a specific population. The pre-dilution and RRT strategy was associated with a dilution of analytes. However, the percentage of predilution was similar between groups and we apply a systematic correction for predilution. The clinical severity appeared not to be a sufficient criterion to select patients with significant LPS translocation. Approximately one-third of patients had cardiac arrest. This could represent a bias as brain damage and post-cardiac arrest syndrome may influence outcomes. We did not account for potential confounders such as variations in the underlying severity of illness, differences in comorbid conditions, or the timing of intervention initiation relative to the onset of shock. The study did not include long-term follow-up beyond 30 days, which might have provided additional insights into the potential benefits or drawbacks of the OXIRIS filter in this patient population. Assessing renal function beyond day 28 and evaluating its evolution in the subsequent weeks or months would have been valuable for understanding the long-term impact of the intervention on renal health. We measured 3OH fatty acids, thus, part of 3OH fatty acids measured might be human metabolites. Nevertheless, excluding non-esterified 3OH-FA reduces the representation of human metabolites among 3OH-FA. Due to inter-batch variability, we could not compare our data with previous LPS concentrations measured in healthy volunteers which would have help us interpret our results (ie. absence of effect due to low translocation). Despite randomization, the ultrafiltration rate appeared to be higher in the OXIRIS filter group.

## Conclusion

In patients with cardiogenic shock supported by VA-ECMO and requiring renal replacement therapy, we could not evidence any reduction in LPS blood concentration in patients treated with OXIRIS filter in comparison to ST-150 filter. Additionally, we could not evidence any reduction in inflammatory biomarker, lower organ failure, improved vasoplegia or survival. Given the small sample size of our cohort, further investigations are necessary to confirm our findings, explore endotoxemia in the context of cardiogenic shock, and assess the potential role of biomarker-based patient selection for these therapies.

## Supplementary Information

Supplementary material 1.

## Data Availability

Because indirect nominative data cannot be shared publicly under French laws, we cannot upload our minimal underlying data set, the datasets generated and/or analyzed during the current study are not publicly available but are available from the corresponding author on reasonable request.

## Abbreviations

CICU: Cardiac intensive care unit
EDTA: Ethylenediaminetetraacetic acid
ELISA: Enzyme-linked immunosorbent assay
GLP-1: Glucagon like peptide 1
I-FABP: Intestinal fatty acid binding protein
IL: Interleukin
LAL: Limulus amoebocyte lysate
LPS: Lipopolysaccharide
MCP: Monocyte chemoattractant protein-1
RRT: Renal replacement therapy
SOFA: Sequential organ failure assessment
TLR: Toll like receptor
TNF: Tumor necrosis factor
VA-ECMO: Veno arterial extracorporeal life support
