# Supplementary material for: Endotoxin removal by the OXIRIS filter for cardiogenic shock requiring veno-arterial extra-corporeal life support: the ECMORIX randomized controlled trial
Source: Ann Intensive Care. 2025 Jul 8;15:92. doi: 10.1186/s13613-025-01499-z (PMC12238705; doi:10.1186/s13613-025-01499-z)
Supplement: Supplementary file 1 — Supplementary material 1. [file 13613_2025_1499_MOESM1_ESM.docx]

|  | ST-150  N = 20 | Oxiris  N = 20 |
| --- | --- | --- |
| **RRT parameters (H0)** |  |  |
| Qs (ml/h) | 150 [150;200] | 200 [150;200] |
| Q_UF_ (ml/kg/h) | 20.6 [18.1;28.6] | 30.3 [20.6;34.8] |
| Percentage of pre-dilution | 33 [32 ;33] | 33 [33 ;33] |
| Depletion (ml/h) | 0.00 [0.00;42.5] | 0.00 [0.00;0.00] |
| Citrate (n,%) | 15 (75%) | 10 (52.6%) |
| **EC**MO **parameters (H0)** |  |  |
| Output (L/min^1^/m²) | 1.6 [1.5 ;1.8] | 1.6 [1.4 ;1.7] |
| Gaz flow (L/min) | 3.00 [2.00;3.62] | 2.75 [2.50;4.00] |
| Femoral position | 17 (85.0%) | 20 (100%) |

**Sup Table 1.** ECLS and RRT parameters according to group allocation

Qs Blood flow rate; Q_UF_: ultrafiltration rate; ECMO: extra-corporeal membrane oxygenation; RRT: renal replacement therapy

Data are given as median [Q1;Q3] or number (percentage).


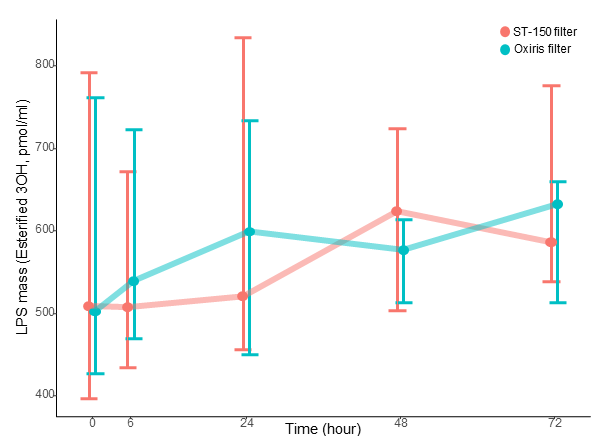


**Sup Figure 1.** Lipopolysaccharides mass kinetic depending on group allocation

Results are represented as median an[Q1;Q3]; there were no significant between group differences by linear mixed modelling.

LPS: Lipopolysaccharides


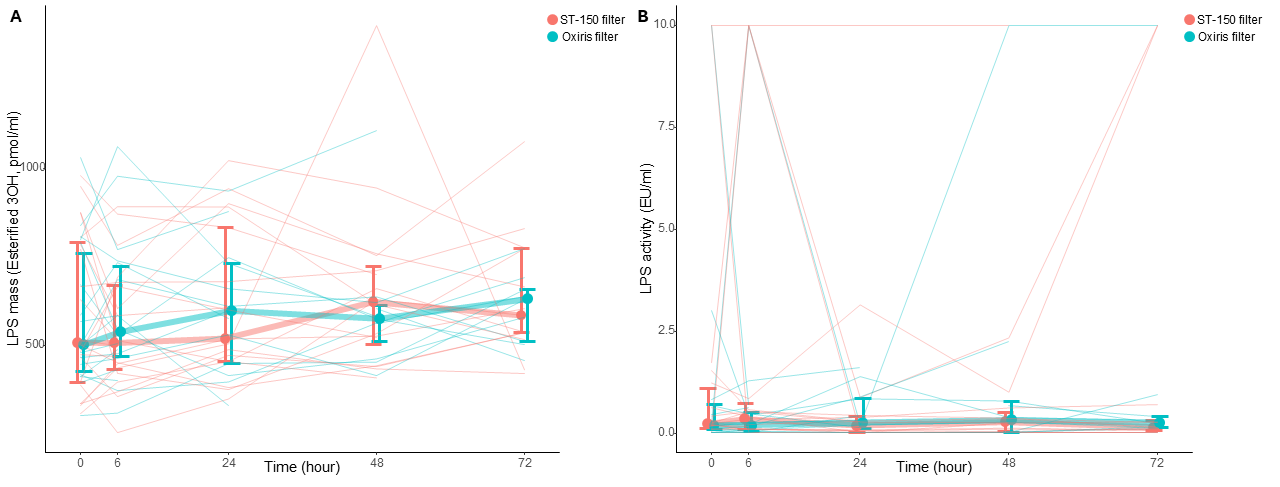


**Sup Figure 2.** Spaghetti plot for LPS mass (A) and LPS activity(B)

Bold lines are median and Q1, Q3


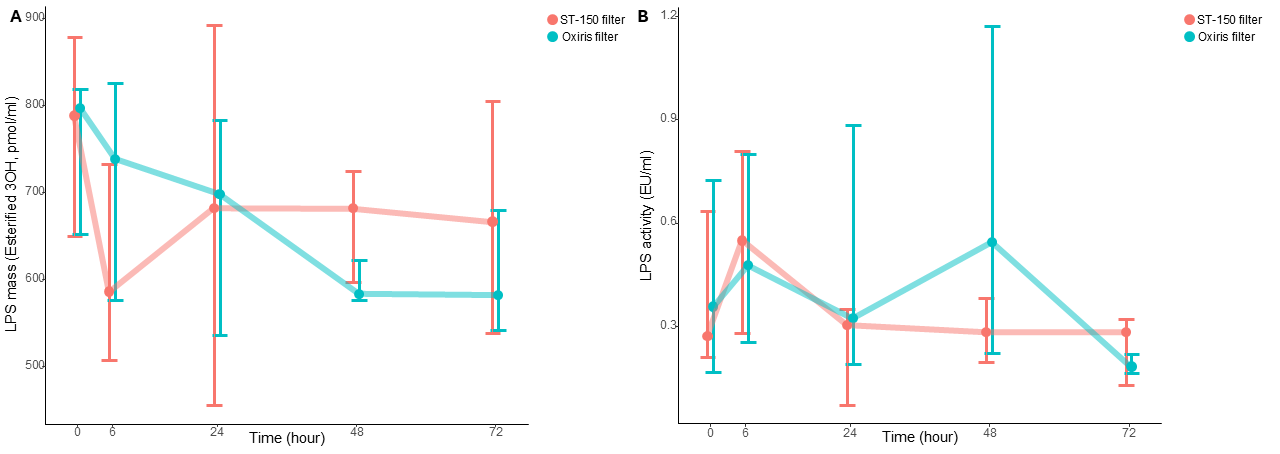


**Sup Figure 3.** LPS mass (A) and activity (B) kinetics according to the group of randomizations in patients with high LPS mass at baseline (8 with OXIRIS filters vs 11 with ST-150 filters).

Results are presented as median (Q1,Q3)
